# Inverse 3D Printing with Variations of the Strand Width of the Resulting Scaffolds for Bone Replacement

**DOI:** 10.3390/ma14081964

**Published:** 2021-04-14

**Authors:** Michael Seidenstuecker, Pia Schilling, Lucas Ritschl, Svenja Lange, Hagen Schmal, Anke Bernstein, Steffen Esslinger

**Affiliations:** 1 G.E.R.N. Tissue Replacement, Regeneration & Neogenesis, Department of Orthopedics and Trauma Surgery, Medical Center—Albert-Ludwigs University of Freiburg, Faculty of Medicine, Hugstetter Straße 55, 79106 Freiburg, Germany; pia.schilling@uniklinik-freiburg.de (P.S.); lucas.ritschl@uniklinik-freiburg.de (L.R.); svenja-lange95@web.de (S.L.); hagen.schmal@uniklinik-freiburg.de (H.S.); anke.bernstein@uniklinik-freiburg.de (A.B.); 2 Institute for Manufacturing Technologies of Ceramic Components and Composites (IMTCCC), Faculty 07, University of Stuttgart, Allmandring 7b, 70569 Stuttgart, Germany; steffen.esslinger@ifkb.uni-stuttgart.de

**Keywords:** FDM, inversely, β-TCP, compressive strength, bone replacement

## Abstract

The objective of this study was to vary the wall thicknesses and pore sizes of inversely printed 3D molded bodies. Wall thicknesses were varied from 1500 to 2000 to 2500 µm. The pores had sizes of 500, 750 and 1000 µm. The sacrificial structures were fabricated from polylactide (PLA) using fused deposition modeling (FDM). To obtain the final bioceramic scaffolds, a water-based slurry was filled into the PLA molds. The PLA sacrificial molds were burned out at approximately 450 °C for 4 h. Subsequently, the samples were sintered at 1250 °C for at least 4 h. The scaffolds were mechanically characterized (native and after incubation in simulated body fluid (SBF) for 28 days). In addition, the biocompatibility was assessed by live/dead staining. The scaffolds with a strand spacing of 500 µm showed the highest compressive strength; there was no significant difference in compressive strength regardless of pore size. The specimens with 1000 µm pore size showed a significant dependence on strand width. Thus, the specimens (1000 µm pores) with 2500 µm wall thickness showed the highest compressive strength of 5.97 + 0.89 MPa. While the 1000(1500) showed a value of 2.90 + 0.67 MPa and the 1000(2000) of 3.49 + 1.16 MPa. As expected for beta-Tricalciumphosphate (β-TCP), very good biocompatibility was observed with increasing cell numbers over the experimental period.

## 1. Introduction

Diseases and defects of the skeletal system continue to increase in our society. The reason for this is the increasing average age of the population [1]. According to the Federal Statistical Office, 193,759 knee endoprostheses and 243,477 hip endoprostheses were implanted in Germany in 2019 [2]. In comparison, only 128,932 knee arthroplasties, as well as 194,453 hip arthroplasties, were implanted in 2004 [3]. In terms of knee arthroplasties, this corresponds to an increase of one third and one fifth for hip arthroplasties within 15 years. With the increasing importance of endoprostheses for the skeletal system, the manufacturing processes are also changing. The treatment of other diseases of the skeletal system is also evolving. In addition, artificial bone substitutes are playing an increasingly important role due to their rapid availability. Wide ranges of different ceramic materials are used. Zirconium oxide ceramics, for example, are used for the heads of hip joint implants due to their high strength [4,5]. Other zirconium-based ceramics such as zirconium dioxide are used as tooth replacement materials for inlays, crowns [6] or post teeth, for example [4]. Biodegradable ceramics and bioglasses are also used. Bioglass [7], biosilicates (like calcium silicate) [8,9] or calcium-based ceramics (e.g., calcium sulphates [10], calcium phosphates [11]) are used as coatings for implants to achieve better ingrowth of the bone. In addition, these calcium sulfate or calcium phosphate ceramics can also be used as a material for bone cements [12] and in block form as bone wedges [13] in the treatment of bone defects (especially critical size defects that cannot be bridged by bone alone). Hydroxyapatite (HA) [14], as well as alpha- and beta-tricalcium phosphate ceramics [15,16], are already used in pure form or as a composite with other biodegradable ceramics [17] in clinical practice. Due to the porous structure of the ceramics, they can also be used as drug delivery devices [18] in addition to their pure support function during bone healing. Thus, drug-coated ceramics [19] are described in the literature, as well as composites of hydrogels introduced into the ceramics [20] to achieve a delayed drug release. Other approaches are also being pursued by adding the ceramics as powder or granules to scaffolds made of hydrogels [21] or xerogels, e.g., collagen [22,23]. This offers the advantage of being able to print these gels by means of 3D extrusion. There are also commercially available pastes made of calcium phosphate that can be printed or injected into the defect and set on contact with water [24,25]. Traditional arthroplasty manufacturing such as casting processes followed by reprocessing is adapting with the advent of 3D printing processes. Tissue engineering, especially for hard tissues such as bone, has been given a number of new opportunities by the development of 3D printing [26]. Tissue engineering of bone tissue is a complex endeavor [1], where the difficulty lies in the production of complex structures at a necessary scale, especially for human applications [27]. Therefore, it is imperative that the materials used are biodegradable and have varying porosity [1,28]. The main purpose of 3D printed constructs is to provide mechanical support during the bone repair or regeneration process. Bose et al. [1] added growth factors and drugs to such 3D printed scaffolds to cause faster bone healing. Bone tissue is a complex tissue. The requirements for 3D printed scaffolds to be used to treat bone defects are equally complex. They must: be biocompatible, have similar mechanical properties, and comparable pore size [29]. Three-dimensional printing can be divided into different processes. First, there is powder-based 3D printing (which we described in a previous paper [30]), where a binder is printed into a powder bed. Another option would be selective laser sintering (SLS), in which the powder is selectively melted with a laser [31]. In this case, the particle size of the powder is the limiting factor for the size of the constructs. Another technique is 3D plotting, in which a ceramic cement, e.g., oil-based, is printed or placed in an aqueous solution. Upon contact with water, the setting reaction starts and the construct solidifies [24]. The fused deposition modeling (FDM) process is another of these 3D printing processes, first described by Crump et al. [32] and has been an integral part of 3D printing research and development ever since. The FDM process is a typical approach that uses heat to produce ceramic scaffolds [29,33]. Advantages of the FDM process are the low costs and that almost arbitrarily complex geometric structures can be fabricated [34,35]. The ceramic model is fabricated with a framework made of a thermoplastic material. For our work polylactide (PLA) was used. The scaffold provides an inverse shape and structure for the pore system of the framework. That is, the PLA is used as a “sacrificial template” into which the ceramic, in our case β-TCP, is cast [36]. The basis for 3D printing the PLA framework is a computer-aided design drawing (STL file), which is then printed using the FDM process. Calcium phosphates (CaP) are mainly used for biomedical applications [37]. CaP are a non-toxic biomaterial and they do not cause foreign body reactions [29,34,38]. Since all previous studies have only printed 3D scaffolds into which bone is then arbitrarily grown, this study, like the previous one [39], focuses on inverse printing. The aim of this work was to vary the wall thickness and pore structure within a 3D construct and to investigate the influence on the strength as well as the degradation behavior. To this end, as before, the FDM process was used to produce the sacrificial structures. These were again made from PLA, as it is also a biomaterial and burns at low temperatures (compared to the sintering temperatures of CaP).

## 2. Materials and Methods

### 2.1. Sample Manufacturing

**Figure 1:**
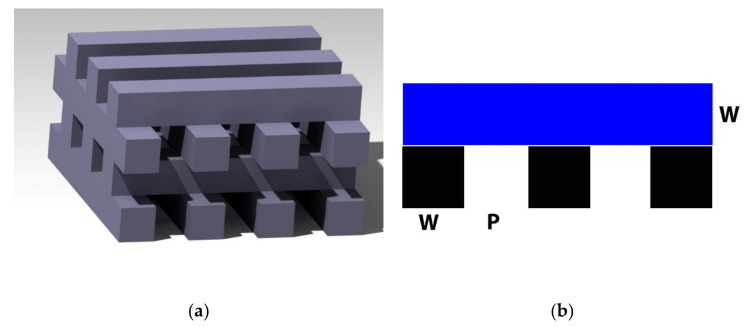
β-TCP Scaffolds; (**a**): 3D rendering from CATIA V5R19 (Dassault Systems, Vélizy-Villacoublay, France); (**b**): schematic sketch, W corresponds to the wall thickness of the sintered scaffold, which was varied from 1500–2500 µm, P corresponds to the pore size, which was varied from 500–1000 µm.

**Figure A1:**
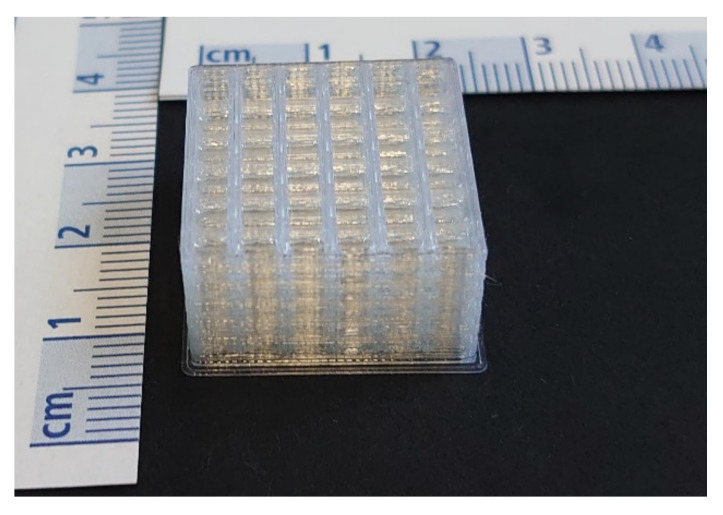
Exemplary Image of inversely 3D printed sacrificial structure which represents the pore structure within the final scaffold.

We describe the fabrication principle of the 3D printed CaP molded bodies used for this project elsewhere [39,40,41]. A PLA mold was made using the FDM process on a Prusa i3 MK3S+ 3D printer (Prusa Research, Prague, Czech Republic). This sacrificial structure represents the negative (i.e., internal pore structure) of the final ceramic framework. The final ceramic molded body was fabricated using a slip casting process, in which the ceramic slip was poured over the plastic mold. A 0.5 mm nozzle was used on the Prusa i3 MK3S+ to produce the PLA molds with strand widths of 500 and 1000 µm. A 0.4 mm nozzle was used to produce the PLA mold with the strand width of 750 µm. The diameter of the commercially available PLA filament (3DJake, niceshops GmbH, Paldau, Austria) was 1.75 mm. To avoid warping of the printed structures, the print bed was heated to 60 °C which is close to the glass transition point of PLA. The nozzle temperature was set to 215 °C. The layer thickness was 250 µm for each model. The strand spacing of the three different patterns was varied from 1500 to 2500 µm. To obtain the final bioceramic scaffolds, a water-based slurry was filled into the PLA molds. The composition of the ceramic slurry was: 70 wt% β-TCP (Chemische Fabrik Budenheim, Budenheim, Germany) and 1 wt% based on solids content DOLAPIX CE64 (Zschimmer and Schwarz, Lahnstein, Germany) as dispersant. Here, the particle sizes of the β-TCP powder ranged from 0.6 to 40 µm (d10 = 2.0 ± 0.04 µm; d50 = 5.27 ± 0.08 µm and d90 = 14.84 ± 0.09 µm). The PLA molds were placed on a porous gypsum board onto which the slurry was applied using the conventional slip casting method. This created capillary forces that caused the water to be removed and the remaining ceramic particles to compact slightly during this process. As a result, the ceramics became denser and progressively stronger [42]. Subsequently, drying was carried out for 24 h on the gypsum board. After that, the PLA sacrificial molds (see Figure A1 Appendix A) were burned out at about 450 °C for 4 h (Nabertherm N200 H, Nabertherm GmbH, Liliental, Germany) [40]. The resulting green bodies already exhibited the defined pore sizes (depending on the PLA mold used). Finally, to give the model mechanical strength and density, the samples were sintered at 1250 °C. For this purpose, the temperature was first heated to 1000 °C at 125 K/h and then to 1250 °C at 100 K/h. This temperature was maintained for 4 h and then cooled down again with the same temperature steps [42,43]. According to this procedure, samples with a strand width of 500, 750 and 1000 µm were produced with the 3D printer, filled with ceramic slurry and sintered. In the following, the designations 500, 750, and 1000 µm remain, which now refer to the empty spaces between the β-TCP strands to differentiate the samples. These inter-strand voids were varied in this experiment: 1500, 2000, and 2500 µm (see Figure 1).

### 2.2. Sample Characterization

#### 2.2.1. Dimensions

A Burg-Wächter PS 7215 digital caliper (Burg-Wächter, Wetter-Volmarstein, Germany) was used to determine the dimensions of the specimens. Olympus SZ61 stereo microscope (Olympus, Shinjuku, Japan) and KEYENCE VK-X210 3D scanning microscope (Keyence, Osaka, Japan) were used to determine the strand width and pore size of our inversely 3D printed ceramics. The voids within the ceramics were measured using Image-J software (Fiji version 1.52 h). The macroporosity of our samples was calculated from the measured values for strand width and pores using the following equation:

$$\mathrm{Macro}\;\mathrm{porosity}\;\left[\%\right]=\frac{\mathrm{pore}\;\mathrm{volume}\;\left[{\mathrm{mm}}^{3}\right]}{\mathrm{scaffold}\;\mathrm{volume}\;\left[{\mathrm{mm}}^{3}\right]}\times 100$$

#### 2.2.2. Surface Roughness

The surface roughness parameter (Sa) was determined for the different scaffolds using 3D laser scanning microscope Keyence VK-X250 (Keyence, Osaka, Japan). For this purpose, at least 3 different samples of each size (see Table 1) were examined at room temperature and 1000× magnification. On each sample, at least 5 different positions were examined in terms of surface roughness.

#### 2.2.3. Mechanical Testing

A Zwick Z005 universal testing machine (Zwick, Roell, Ulm, Germany) with a load cell for 5 kN was used for the mechanical tests. The tests were performed as described elsewhere [30,39] with a preload of 1 N, displacement-controlled up to the break or deformation of 50%. At least 10 different samples (with and without incubation in SBF) were measured from each scaffold. All measurements were repeated 3 times.

#### 2.2.4. Microstructure and Elemental Analysis

The pore structure within the ceramic was determined using ESEM FEI QUANTA 250 FEG (FEI, Hillsboro, OR, USA) with an Oxford EDX (energy dispersive X-ray spectrometer) instrument (Oxford Instruments, Tubney Woods, UK). For this purpose, the samples were cut in the middle (with a razor blade, Apollo Herckenrath GmbH & Co, Solingen, Germany), any spalling was removed using compressed air, glued to the pin sample holders with a double-sided carbon conductive pad (Plano GmbH, Wetzlar, Germany), and fixed in the microscope in the sample holder. ESEM images were acquired with an accelerating voltage of 10 kV. In addition, EDX measurements were performed to determine the elemental composition. EDX measurements were performed at room temperature, with a 5 min lifetime corrected measurement and an excitation voltage of 20 kV. In addition, XRD analysis of a selected sample was performed using the Bruker D8 Advance (Bruker, Billerica, MA, USA). Prior to analysis, the sample was ground using an agate mortar. Measurement conditions were Bragg-Brentano geometry, equipped with Cu anode and secondary graphite monochromator, scintillation counter, 40 kV/40 mA, 1°2- theta/min, step size 0.02°2theta. Profex version 4.3 was used for the Rietveld refinement analysis of the XRD data.

### 2.3. Biocompatibility

All experiments were performed using MG-63 cells (ATCC CRL 1427). Cells were first thawed in passage 15 from the liquid nitrogen tank (at −196 °C). For this purpose, they were incubated in Dulbecco’s Modified Eagle Medium (DMEM) containing F12 nutrient and the additions of 1% penicillin/streptomycin (P/S, Sigma Aldrich (now Merck), Darmstadt, Germany) and 10% fetal bovine serum (FBS, Merck, Darmstadt, Germany) in a New Brunswick Galaxy 170R incubator (Eppendorf, Hamburg, Germany) at 37 °C and a CO_2_ saturation of 5%. Splitting of cells was performed twice a week at 1:10 and 1:5. All scaffolds were heat sterilized at 200 °C for 4 h in a UF500 drying oven (Memmert, Schwabach, Germany) and then used for biocompatibility testing and experiments with SBF. The biocompatibility of β-TCP has been demonstrated by us in previous studies [20,30,39,44]. For this reason, we did not determine cell proliferation and lactate dehydrogenase in this work, but only performed live/dead assay using MG-63 cells.

#### Live/Dead Assay

Live/dead assays in this work were performed at 3, 7, and 10 days. For each time point, at least three samples per scaffold size were placed in the cell culture plates (Greiner Bio-One International GmbH, Kremsmünster, Austria). Altogether, 50,000 cells suspended in 200 µL medium were added directly to the samples and incubated for 2 h at 37 °C and a CO_2_ saturation of 5%. The reason for this is to allow cellular attachment to our samples. After these two hours, the samples were incubated in 2.5 mL each of DMEM-F12 (part no. BE12-719F, Lonza, Basel, Switzerland) complete medium for the predefined periods (3, 7, 10 days). At the defined times (3, 7, 10 days), the samples were stained. The staining solution was prepared by adding 2 mL DPBS (art. no. 14190-094, Gibco, Grand Island, NE, USA) to a Falcon tube (Greiner Bio-One International GmbH, Kremsmünster, Austria) and 4 µL ethidium homodimer III (Eth D-III) solution (together with calcein part of the Live/Dead Cell Staining Kit II (PromoCell, Heidelberg, Germany)) according to the manufacturer’s protocol (PromoCell). An amount of 1 µL of calcein dye was added after mixing the staining solution. All steps were performed in the dark to avoid photobleaching of staining solution and samples. To eliminate serum esterase activity, all samples at a time point had the medium removed and the cells washed. Staining was then performed according to a previously published protocol [19]. Evaluation was performed using an Olympus fluorescence microscope (BX51, Olympus, Osaka, Japan) at five different positions on the samples at 5× and 10× magnification. Subsequently, the samples were first cut vertically (razor blade), examined at three different positions with the same 5× and 10× magnifications, and then cut horizontally and viewed at the same three positions with the aforementioned magnifications. Live cells fluoresce green under blue light, while dead cells fluoresce red. Previous studies have shown that β-TCP is biocompatible [20,30,45,46]. Therefore, in this experiment, we omit the determination of cell proliferation and lactate dehydrogenase and restrict ourselves to live/dead determination using MG-63 cells.

### 2.4. Incubation in Simulated Body Fluid (SBF)

The SBF solution was prepared by adding the chemicals described in Table 2 according to the indicated order in 500 mL distilled water (according to Jalota et al. [47]).

To avoid any microbial growth in the solution, it was sterilized through a 0.2 µm membrane filter (Brand, Wertheim, Germany). Five scaffolds of each condition were placed in a 12-well cell culture plate and covered with 3.5 mL of the SBF solution. Incubation was then performed for 28 days at 37 °C and 5% CO_2_ saturation. After incubation for 28 days, the SBF solution was removed and the samples were washed at least three times with distilled water. Drying was then carried out at 40 °C.

### 2.5. Statistics

All data are presented as means ± standard deviation. Measured values were also analyzed using one-way analysis of variance (ANOVA) with a significance level of *p* < 0.05. Origin 2021 Professional SR1 (OriginLab, Northampton, MA, USA) was used for all statistical analyses.

## 3. Results

### 3.1. Sample Characterization

#### 3.1.1. Dimension

**Figure 2:**
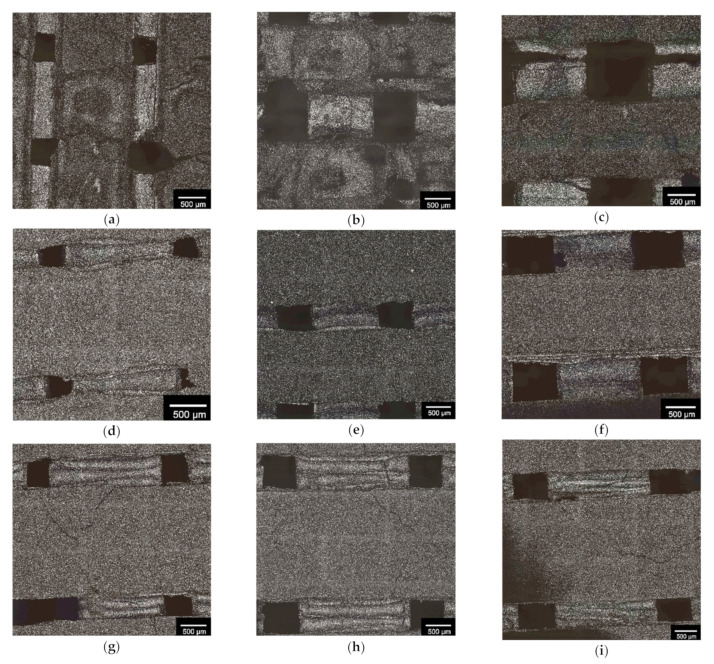
Overview of 3D printed scaffold geometries: (**a**) 500(1500); (**b**) 750(1500); (**c**) 1000(1500); (**d**) 500(2000); (**e**) 750(3000); (**f**) 1000(2000); (**g**) 500(2500); (**h**) 750(2500); (**i**) 1000(2500): where the first number corresponds to the pore size and the second number corresponds to the strand width; white bar = 500 µm; images captured with KEYENCE 3D laser scanning microscope VK-X210.

The different inverse 3D printed β-TCP scaffolds were measured several times. As in previous studies [39], the samples with 500 µm pore size were found to have the smallest dimensions compared to the samples with larger pores (750 and 1000 µm). However, depending on the strand widths (see Figure 2), the measured sizes within the group with the same pore size increased as expected. Table 3 summarizes the results for the dimensional measurements of the samples.

**Figure 3:**
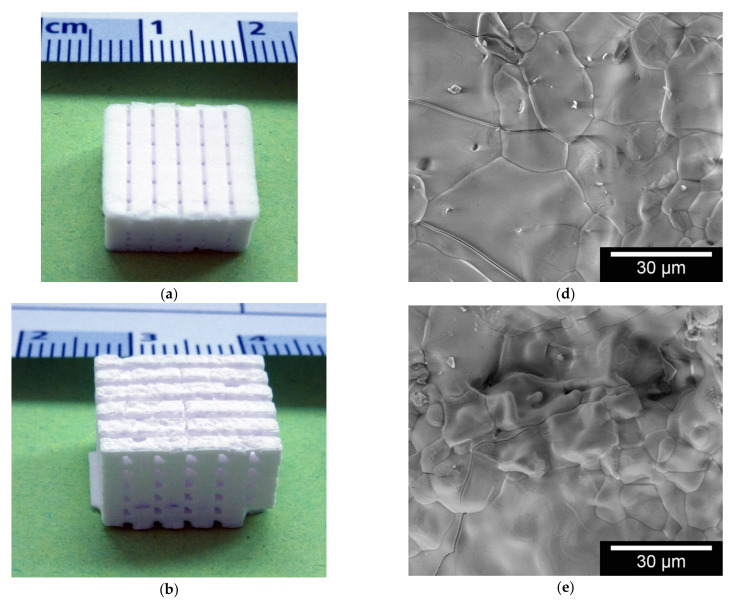

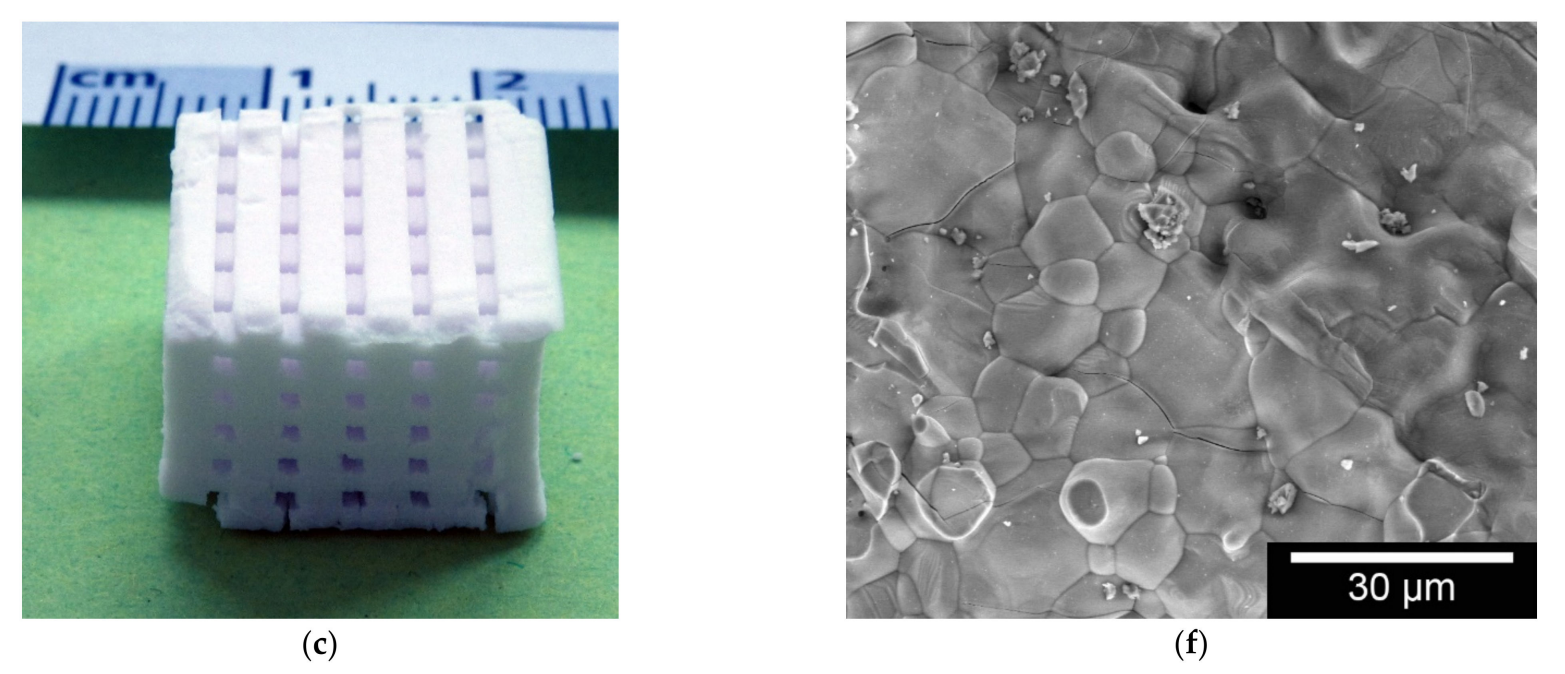
Macro and microstructure of the scaffolds; exemplary images of band width 2000 µm three different pore sizes: (**a**) 500 µm; (**b**) 750 µm; and (**c**) 1000 µm; microstructures taken with FEI QUANTA 250 FEG, 20 kV, 3200× magnification (HFW 93.3 µm) of the sample surface: (**d**) 500 µm; (**e**) 750 µm; and (**f**) 1000 µm.

The pore size and strand widths of the scaffolds were analyzed using a Keyence 3D laser scanning microscope (VK Analysis, version 3.5.0.0; Keyence, Osaka, Japan). This allowed sintering shrinkages of the scaffolds of 8.3 ± 0.8 to 22.6 ± 0.3% to be detected. An overview of the measured pore sizes and strand widths (see Figure 3), as well as the determined average sinter shrinkage, is shown in Table 4.

#### 3.1.2. Microstructure and Elemental Analysis

**Figure 4:**
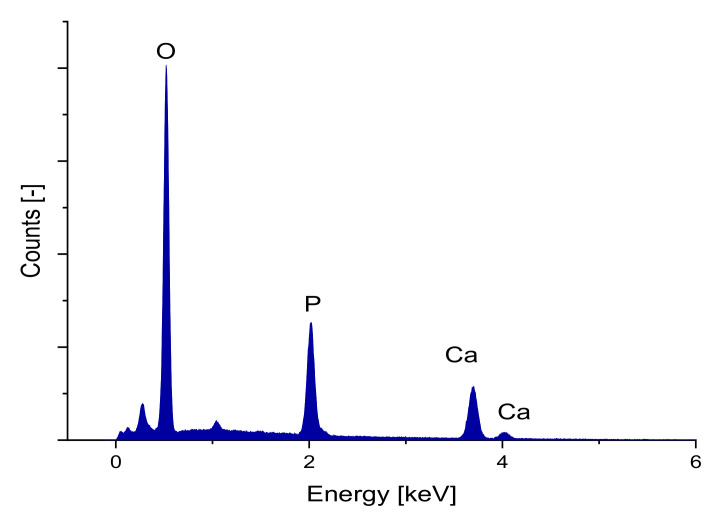
EDX spectrum of a sample, taken with an FEI Quanta ESEM FEG 250 FEG and an Oxford EDX unit, 10 kV acceleration voltage, 30 min counting period live time corrected.

To determine the elements in the sample, elemental analysis of the grape-like surface structure on the various samples was performed using EDX. Figure 4 shows an example of the EDX spectrum of the 1000(2000) µm samples. Table 5 shows the percentages for relevant atoms for that sample.

The Ca/P ratio of the sample was determined to be 1.5 indicating that the scaffolds produced consisted of β-TCP [28,48].

**Figure 5:**
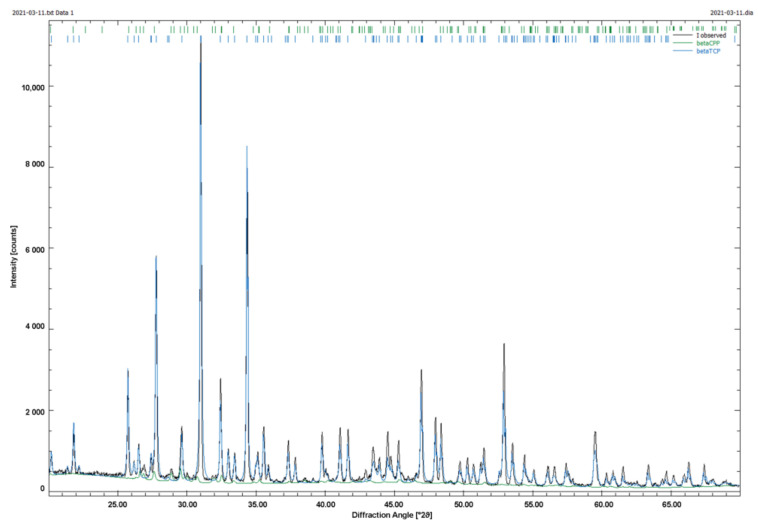
XRD Pattern of 3D printed samples, as example 1000(2000).

Our samples were all prepared using the same sintering protocol. Therefore, we limited ourselves to the XRD analysis of one sample (1000(2000)). The XRD pattern is shown in Figure 5 a subsequent Rietveld refinement analysis showed that the sample is composed of 99% β-TCP and 1% Calcium pyrophosphate (CPP). The CPP still originates from the manufacturing process. Carbon-containing compounds (originating from the sacrificial structures or the binder) could not be detected (see Figure 5).

#### 3.1.3. Surface Roughness

**Figure 6:**
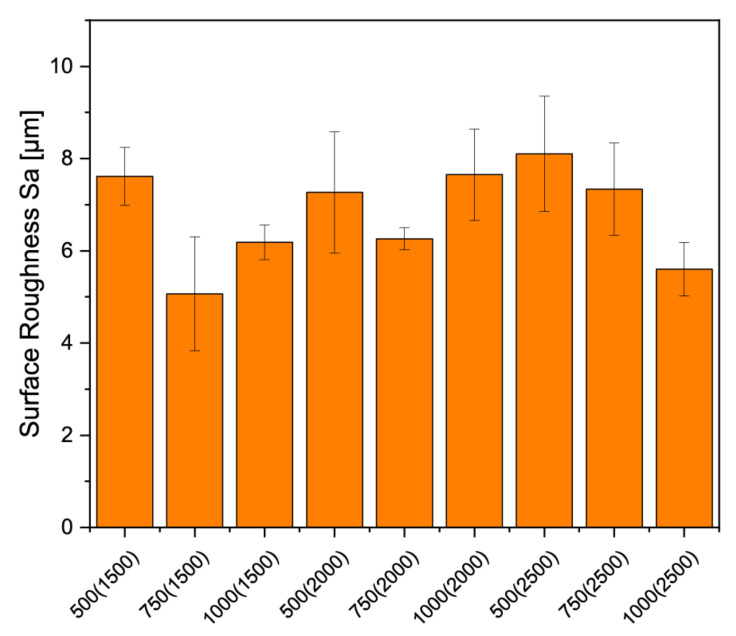
Overview of the surface roughness of the different scaffolds, the measurements were carried out on the Keyence 3D laserscanning microscope VK-X210.

In order to determine the surface roughness, 5 separate localized areas on the surfaces of 3 specimens for each scaffold condition were examined. The roughness parameter Sa was averaged for the different scaffolds from the individual measurements. The mean values are shown in Figure 6. No significant difference could be determined with *p* < 0.05.

#### 3.1.4. Mechanical Testings

The influence of 28 day incubation in SBF was clear for the scaffolds with 1500 µm wall thicknesses. In particular, the samples with 1000 µm pores were affected the most. The 1000 µm sample’s compressive strength was 3.5 ± 1.2 MPa for the untreated samples and reduced to a value of 0.6 ± 0.3 MPa after incubation for 28 days in SBF. For the samples with 750 µm pore size, the difference in compressive strength was not as distinct: the untreated samples had a compressive strength of 5.9 ± 0.8 MPa before incubation which decreased to 3.9 ± 0.5 MPa after incubation. The effect was not as strong for the scaffolds with 2000 µm wall thickness. The samples with 1000 µm pore size showed compressive strength values of 3.5 ± 1.2 MPa. Incubation in SBF decreased this value to 2.9 ± 0.7 MPa. Similar observations were made for the samples with 750 µm pore size where incubation in SBF changed the compressive strength from 6.7 ± 2.6 MPa to 5.1 ± 1.5 MPa. The samples with 2500 µm wall thickness showed a moderate influence. The samples with 1000 µm pore size showed a change in compressive strength by incubation in SBF from 9.3 ± 0.6 MPa to 6.0 ± 0.9 MPa, whereas the samples with 750 µm pore size showed nearly no change: 8.1 ± 2.0 MPa untreated and 7.9 ± 0.8 MPa after incubation in SBF.

**Figure 7:**
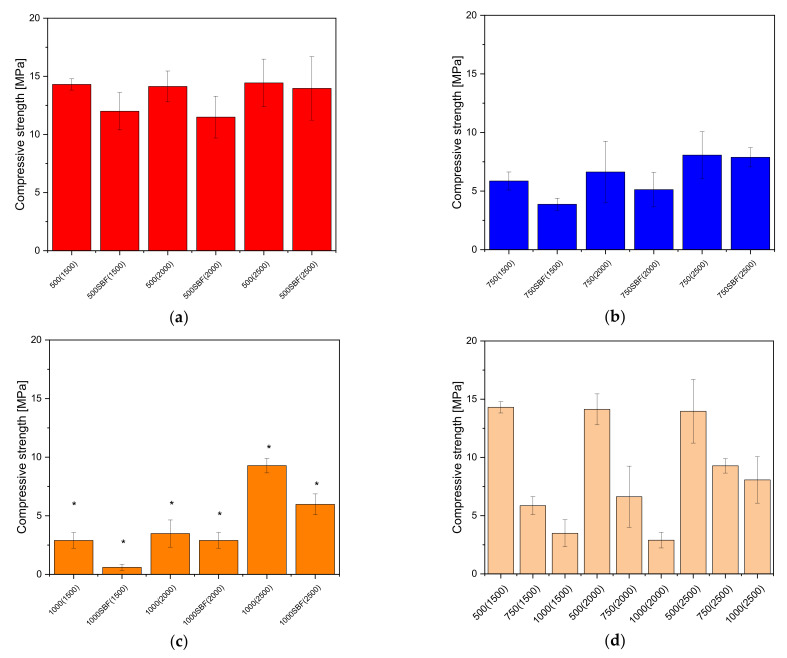
Comparison of the compressive strength of the different inverse 3D printed scaffolds: (**a**) 500 µm pore size; (**b**) 750 µm pore size; (**c**) 1000 µm pore size; (**d**) comparison of the different pore sizes and strand widths; the measurements were performed on Zwick universal testing machine Z005; *—significant difference *p* < 0.05.

Comparing the scaffolds with the same pore sizes to each other, there is no significant difference in compressive strength between the different wall thicknesses for the 500 µm pore size. There is also no significant difference in compressive strength when comparing the samples with and without SBF treatment (see Figure 7a). Comparing the compressive strength of the samples with different wall thicknesses from the scaffolds with 750 µm pore size under one another, there is no significant difference. Except for the sample with 1500 µm wall thickness, there is also no significant difference in compressive strength between the samples with and without SBF treatment (see Figure 7b). For the samples with 1000 µm pore size significant differences (*p* < 0.05) were found in compressive strength between different thicknesses, as well as between samples with and without SBF treatment (see Figure 7c).

### 3.2. Biocompatibility

#### Live/Dead Assay

**Figure 8:**
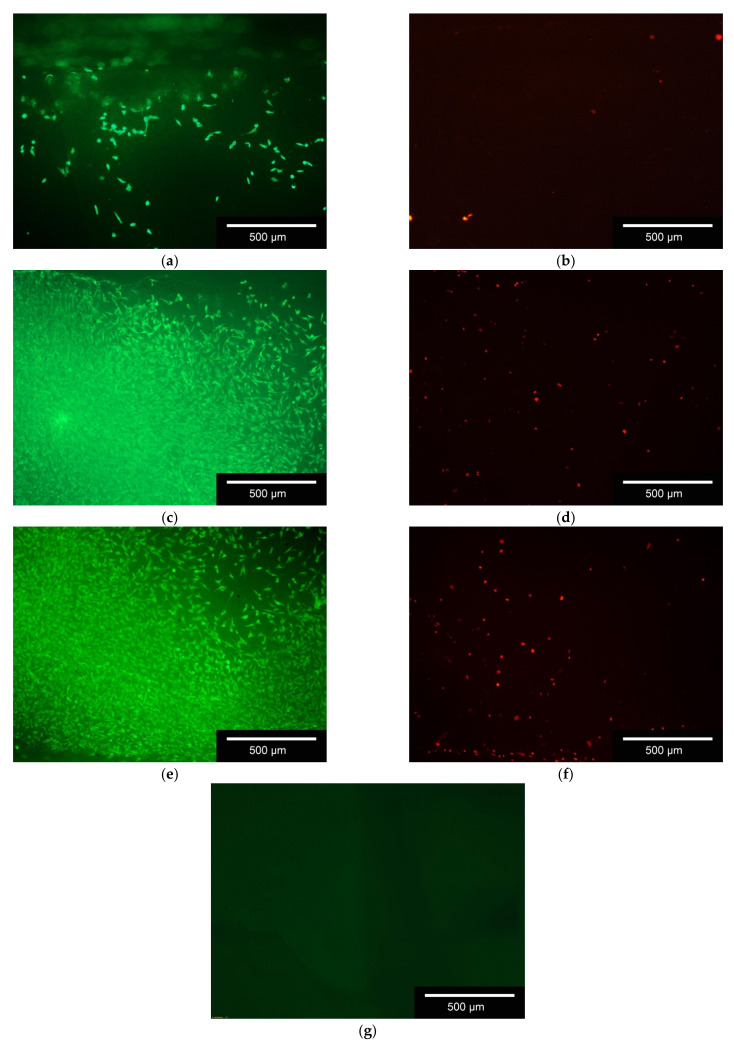
Live/Dead Staining; exemplary images of 1000(2500); (**a**) living cells on the scaffold after 3 days; (**b**) dead cells on the scaffold after 3 days; (**c**) living cells on the scaffold after 7 days; (**d**) dead cells on the scaffold after 7 days; (**e**) living cells on the scaffold after 10 days; (**f**) dead cells on the scaffold after 10 days; (**g**) auto-fluorescence of the ceramics, exposure time 10× as high as the other; living cells = green; dead cells = red; white bar = 500 µm, images taken with Olympus BX-53 Fluorescence microscope.

**Figure A2:**
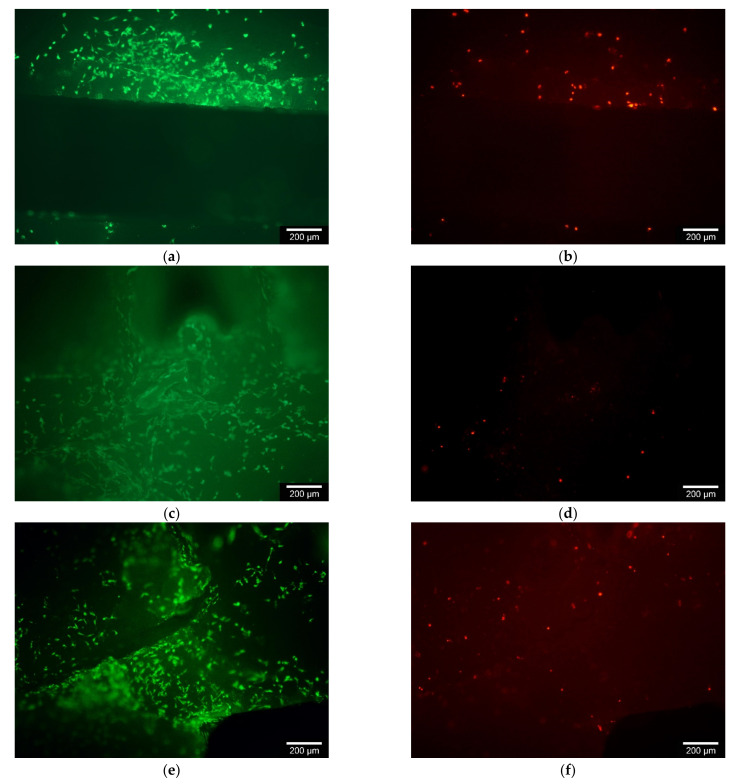
Exemplary Images of Live/Dead Staining 1000(2000) inner surface; (**a**) living cells after 3 days; (**b**) dead cells after 3 days; (**c**) living cells after 7 days; (**d**) dead cells after 7 days; (**e**) living cells after 10 days; (**f**) dead cells after 10 days.

The MG-63 cells were counted by using Image-J (Fiji, Version 1.52 h), through which the cell number/mm^2^ and the percentage of living cells were determined. Figure 8 shows exemplary the live/dead staining of the outer surface of the 1000(2500) µm scaffolds after 3, 7 and 10 days. The inner surface images are included in the Apendix, exemplary for 1000(2000) in Figure A2 Appendix A.

The numbers of living and dead cells per mm^2^ over all test days are shown in Table 6. Different locations of the scaffolds (outer surface, inner surface) were examined. The number of cells per mm^2^ increased continuously over the course of 10 days.

**Figure 9:**
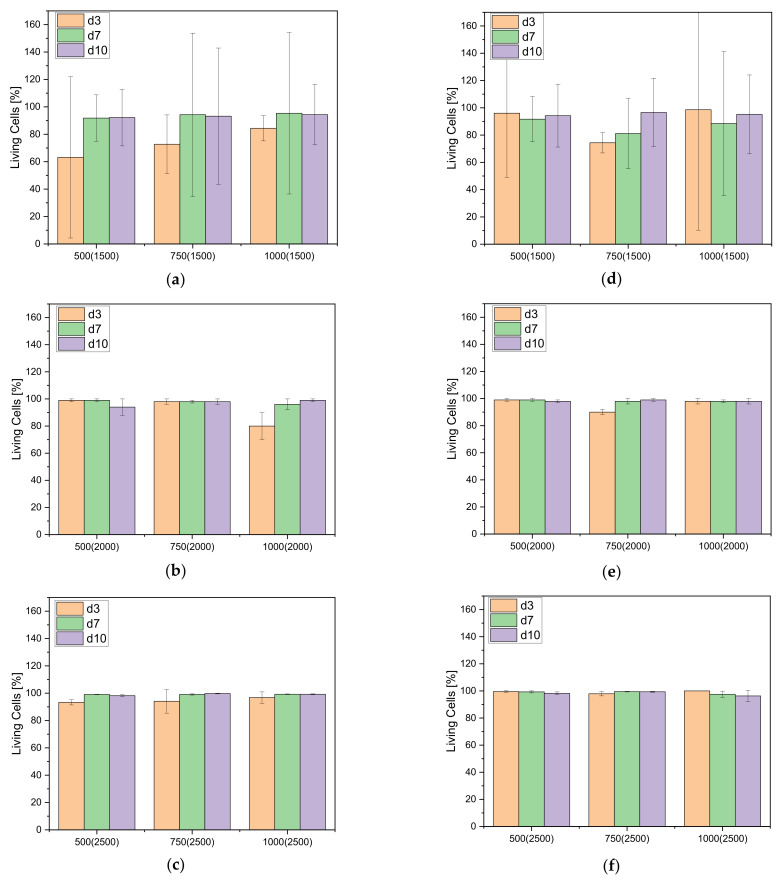
Living Cells in percent for the different band widths after 3, 7 and 10 days; Overview for the (**a**) 1500 µm band width; outer surface; (**b**) 2000 µm band width, outer surface; (**c**) 2500 µm band width, outer surface; (**d**) 1500 µm band width, inner surface; (**e**) 2000 µm band width, inner surface; (**f**) 2500 µm band width, inner surface.

The percentage of living cells on the different scaffolds (inside and outside) is above 95% with a few exceptions (cf. Figure 9).

## 4. Discussion

### 4.1. Sample Characterization

#### 4.1.1. Dimensions

Mehdikhani et al. [49] reported a temperature-dependent linear sintering shrinkage of β-TCP with 14.58% at a sintering temperature of 1100 °C. Khiri et al. [50] reported a sintering shrinkage of 11.52% for HA and β-TCP. In a similar range, the sintering shrinkage of our inverse 3D printed scaffolds had a mean of 12.6 ± 5.0%.

#### 4.1.2. Surface Roughness

Since the inverse 3D printed scaffolds are all based on the same β-TCP powder, the surface roughness is in the same order of magnitude for all of them. The ESEM images of the surfaces of the different scaffolds look very similar due to the similar surface roughness.

#### 4.1.3. Mechanical Testing

As shown in previous work [39], the mechanical characteristics depend on the pore size rather than the wall thickness. The scaffolds with the pore size of 500 µm varied only slightly in compressive strength from 14.3 ± 0.5 MPa for the 500(1500); 14.1 ± 1.3 MPa for the 500(2000) and 14.4 ± 2.0 MPa for the 500(2500). Notably, the 500 and 750 µm pore size scaffolds showed no significant difference in compressive strength for the three different wall thicknesses (1500, 2000, and 2500 µm). Additionally, the difference in compressive strength between the scaffolds without SBF and incubated in SBF was not significant for the 500 and 750 µm pore size. However, there was still a trend that the scaffolds incubated in SBF had lower mechanical stability. The scaffolds with the 1000 µm pores showed a significant difference between the three different pore sizes. Furthermore, for the scaffolds with 1000 µm pores, there was a significant difference with *p* < 0.05 between the scaffolds incubated without or in SBF. Moreover, there is a significant difference between the different pore sizes with the same wall thickness. In their investigations of cylindrical TCP scaffolds, Bose et al. [51] demonstrated that compressive strength decreased with increasing pore size. The samples of Bose et al. [51] showed a slightly larger porosity with values between 29–44%, whereas our samples showed a pore volume of 1227% (see also Table 3). Just like our previous project, the scaffolds investigated in this work show significantly higher compressive strength values in the order of 14 MPa for the samples with 500 µm pore size, and values in the range of 3 MPa for the samples with 1000 µm pore size, compared to the values of Bose et al. [51] with 0.2 MPa. The compressive strength values are all in the range of 2–20 MPa for cancellous bone [52]. Moreover, the specimens with higher wall thickness showed microcracks in the images from ESEM and 3D laser scanning microscope, respectively. According to Zhang et al. [53] who also found microcracks in their work, the reason is the sintering temperature. With increasing sintering temperature, the size of the cracks increased and the compressive strength decreased. In our work, we were also able to detect cracks, but the compressive strength decreased only due to the larger pores, but not due to the cracks (cf. Figure 7d), since these had the same cracks and were sintered at the same temperature (1250 °C). Comparing the compressive strength of the inversely printed scaffolds with those we had produced in previous work using 3D powder printing [30], the values of the inversely printed scaffolds are significantly higher.

### 4.2. Biocompatibility

Similar to our previous work [30], more cells were found on the outer surface of the scaffolds than within the structure. This is due to the cells being placed on the outside of the scaffolds with a pipette as a droplet and as the ceramics are similarly hygroscopic, as shown in the previous work [39], the droplet is then absorbed into the ceramic by capillary forces. Hardly any cells were found outside the ceramics on the surface of the cell culture plates used.

## 5. Conclusions

It is demonstrated that different strand spacing and pore sizes can affect the stability of β-TCP scaffolds. The 500 µm scaffolds have the highest compressive strength of all three pore sizes. They show compressive strength comparable to that of cancellous bone. The 500 µm scaffolds are therefore most suitable for use in bone replacement. Samples with a 500 µm pore size showed no significant difference in compressive strength due to wall thicknesses or SBF degradation. It was also shown that wall thickness only affected sample stability for samples with a pore size of 1000 µm or greater. Variations in strand spacing, as well as pore size, had no influence on biocompatibility. Almost identical cell growth was observed for all three sizes. Since the biocompatibility of β-TCP was determined in many previous projects, the biocompatibility was only determined using live/dead cell staining.

## Figures and Tables

**Table 1:** Overview of the used samples.

Pore Size (µm)	Strand Width (µm)		
	1500	2000	2500
500	500(1500)	500(2000)	500(2500)
750	750(1500)	750(2000)	750(2500)
1000	1000(1500)	1000(2000)	1000(2500)

**Table 2:** Simulated body fluid (SBF) receipt according to Jalota et al. [47].

Chemical Substance	Quantity (g)	Order No. Sigma Aldrich *
NaCl	3.274	S9888
NaHCO_3_	1.1134	S6014
KCl	0.187	P3911
Na_2_HPO_4_ 2H_2_O	0.089	71643
MgCl_2_	0.071	M8266
CaCl_2_ 2H_2_O	0.184	223506
Na_2_SO_4_	0.0355	239313
(CH_2_OH)_3_CNH_2_	3.0285	T1378
1M HCl solution	until pH 7.4	1090571000 **

* Sigma Aldrich (now Merck), Darmstadt, Germany; ** Supelco, Bellefonte, PA, USA.

**Table 3:** Comparison of the dimensions of the different scaffolds.

Sample	Width (mm)	Height (mm)
500(1500)	13.1 ± 0.2	5.6 ± 0.4
750(1500)	13.3 ± 0.1	6.4 ± 0.3
1000(1500)	14.7 ± 0.1	8.1 ± 0.5
500(2000)	13.2 ± 0.2	5.4 ± 0.1
750(2000)	14.1 ± 0.1	8.1 ± 0.1
1000(2000)	15.3 ± 0.1	10.8 ± 0.1
500(2500)	15.6 ± 0.2	5.7 ± 0.1
750(2500)	17.3 ± 0.5	8.3 ± 0.2
1000(2500)	17.5 ± 0.5	9.1 ± 0.3

**Table 4:** Overview of pore size and strand widths of inverse printed 3D constructs.

Sample	Dimensions (µm)		Macro Porosity (%)	Mean Sinter Shrinking (%)
	Pore Size	Strand Width		
500(1500)	456.8 ± 33.7	1384.0 ± 40	21.57	8.3 ± 0.8
500(2000)	388.0 ± 27.2	1544.0 ± 42.4	11.62	22.6 ± 0.3
500(2500)	481.3 ± 30.0	2144.0 ± 12.7	14.90	9.0 ± 7.4
750(1500)	733.3 ± 78.7	1241.0 ± 10	22.05	9.7 ± 10.6
750(2000)	740.0 ± 11.3	1494.5 ± 17.7	26.37	13.3 ± 16.9
750(2500)	675.0 ± 12.5	2255.0 ± 15.6	17.45	9.9 ± 0.1
1000(1500)	914.5 ± 2.1	1298.0 ± 45.3	23.65	11.0 ± 3.5
1000(2000)	864.5 ± 41.7	1498.7 ± 66.7	24.88	19.3 ± 8.1
1000(2500)	870 ± 42.4	2290.5 ± 10.6	26.14	10.7 ± 9.3

**Table 5:** Proportions of relevant elements in the EDX spectrum.

Elements	Atom %
C	8.8
O	58.5
P	13.1
Ca	19.6

**Table 6:** Overview of living/dead cells for the different sized (pores, band widths) scaffolds.

Sample	Day 3		Day 7		Day 10	
	Living	Dead	Living	Dead	Living	Dead
500(1500)	Cells/mm^2^					
Outer Surface	36 ± 33	21 ± 1	180 ± 33	16 ± 8	308 ± 69	26 ± 6
Inner Surface	35 ± 12	5 ± 4	121 ± 20	11 ± 8	164 ± 40	10 ± 4
750(1500)						
Outer Surface	32 ± 9	12 ± 4	197 ± 124	12 ± 8	262 ± 139	19 ± 8
Inner Surface	49 ± 17	3 ± 2	200 ± 84	10 ± 4	218 ± 99	52 ± 23
1000(1500)						
Outer Surface	27 ± 2	5 ± 5	225 ± 139	11 ± 9	265 ± 61	16 ± 11
Inner Surface	54 ± 11	2 ± 1	226 ± 141	40 ± 22	223 ± 44	40 ± 11
500(2000)						
Outer Surface	20 ± 4	2 ± 1	146 ± 121	26 ± 2	231 ± 153	18 ± 8
Inner Surface	56 ± 22	1 ± 1	134 ± 78	5 ± 1	151 ± 82	17 ± 4
750(2000)						
Outer Surface	71 ± 12	17 ± 3	388 ± 286	10 ± 4	441 ± 111	17 ± 11
Inner Surface	51 ± 28	1 ± 1	184 ± 60	3 ± 2	249 ± 140	12 ± 11
1000(2000)						
Outer Surface	63 ± 5	9 ± 3	352 ± 176	18 ± 12	479 ± 124	17 ± 11
Inner Surface	34 ± 10	1 ± 1	251 ± 45	7 ± 1	264 ± 83	6 ± 1
500(2500)						
Outer Surface	126 ± 59	10 ± 5	851 ± 364	8 ± 3	1861 ± 179	35 ± 14
Inner Surface	87 ± 40	1 ± 1	457 ± 121	4 ± 5	532 ± 142	10 ± 6
750(2500)						
Outer Surface	79 ± 51	5 ± 5	821 ± 115	9 ± 5	1803 ± 578	6 ± 6
Inner Surface	89 ± 19	2 ± 2	392 ± 96	2 ± 1	763 ± 372	5 ± 2
1000(2500)						
Outer Surface	105 ± 34	3 ± 3	911 ± 275	8 ± 3	1614 ± 341	12 ± 4
Inner Surface	176 ± 65	0 ± 0	331 ± 75	9 ± 8	471 ± 192	13 ± 13

## Data Availability

The data presented in this study are available on request from the corresponding author.
